# Parathyroid hormone and vitamin D are associated with the risk of metabolic obesity in a middle-aged and older Korean population with preserved renal function: A cross-sectional study

**DOI:** 10.1371/journal.pone.0175132

**Published:** 2017-04-06

**Authors:** Jeonghoon Ha, Kwanhoon Jo, Dong-Jun Lim, Jung-Min Lee, Sang-Ah Chang, Moo I. L. Kang, Bong Yun Cha, Min-Hee Kim

**Affiliations:** Division of Endocrinology and Metabolism, Department of Internal Medicine, College of Medicine, The Catholic University of Korea, Seoul, Korea; University of Alabama at Birmingham, UNITED STATES

## Abstract

**Background:**

In general, obesity is a major contributor to metabolic syndrome (MetS) and is associated with insulin resistance (IR). Metabolically obese but normal weight (MONW) individuals present metabolic abnormalities and features of MetS despite having a normal range of body mass index (BMI). In recent years, different subtypes of obesity have been introduced, including metabolically healthy obese (MHO) and metabolically obese obese (MOO). Also, it has been reported that vitamin D and parathyroid hormone (PTH) are possibly linked with MetS.

**Methods and findings:**

In this study, we aimed to evaluate the association between serum 25(OH)D, serum PTH, and the risk of metabolic obesity in four subtypes using nationally representative survey data for a Korean population conducted between 2008 and 2010. Of the 29,235 Korean participants, 18,997 subjects aged under 50 years were excluded. Participants with diabetes (n = 1,520), renal insufficiency (glomerular filtration rate [GFR] < 45 ml/min/1.73 m^2^, chronic kidney disease [CKD] stage 3b, 4, and 5 according to KDOQI classification [1]) (n = 49), history of treatment for osteoporosis (n = 455), insufficient data (n = 1,613), and fasting time less than 8 hours prior to blood collection (n = 771) were excluded for analysis. Ultimately, 5,830 adults (2,582 men and 3,248 women) were eligible for the present study. And, subtypes of obesity were divided into four types: Metabolically healthy normal weight (MHNW), Metabolically healthy obese (MHO), Metabolically obese but normal weight (MONW), and Metabolically obese obese (MOO). Female subjects with metabolic obesity were more likely to have higher levels of PTH and Male subjects with metabolic health were more likely to have higher serum 25(OH)D levels

**Conclusion:**

We concluded that a positive association between serum PTH concentration and metabolic obesity among female subjects and an inverse relationship between serum 25(OH)D levels and the risk of metabolic obesity were found among male subjects. Further prospective studies are necessary to explore the biological mechanisms underlying these sex-specific findings.

## Introduction

Obesity is a major public health problem, not only in western countries but also in Korea, and its prevalence has increased over the past 30 years. It is known that obesity is a major risk factor for cardiovascular disease, diabetes, and cancer with a consequent reduction in life expectancy [2]. In general, obesity is a major contributor to metabolic syndrome (MetS) and is associated with insulin resistance (IR) [3]. Ruderman *et al*. first suggested that non-obese or slightly obese individuals also presented several risk factors for MetS and those populations are regarded as one end of the spectrum of obesity [4]. Metabolically obese but normal weight (MONW) individuals present metabolic abnormalities and features of MetS despite having a normal range of body mass index (BMI) [5]. Ongoing investigations have established MONW as one of the disease entity requiring serious attention [6]. In recent years, different subtypes of obesity have been introduced, including metabolically healthy obesity (MHO) and metabolically obese obese (MOO). MHO is defined as those individuals who lack any metabolic abnormalities or MetS despite having obesity based on BMI; MOO is characterized by metabolic abnormality or MetS with obesity based on BMI [7].

It has been reported that Vitamin D and Parathyroid hormone (PTH) are possibly linked with MetS [8]. In cross-sectional studies, inverse relationships between serum 25-hydroxyvitamin D (25(OH)D) concentrations and MetS have been noted in several cohorts [9–11]. PTH, which is main regulator of calcium homeostasis along with vitamin D, was also reported to have a positive correlation with IR and the development of MetS [11,12].

In this study, we aimed to evaluate the association between serum 25(OH)D, serum PTH with the risk of metabolically obesity in four subtypes using nationally representative survey data for a Korean population. Because drastic changes are anticipated in PTH and vitamin D status among subjects with chronic kidney disease, we focused on subjects with relatively preserved renal function.

## Methods

### Study population

The study was based on the fifth Korea National Health and Nutrition Examination Survey (KNHANES) 2008–2010, a cross-sectional and nationally representative survey carried out by the Korean Centers for Disease Control and Prevention (CDC). It consists of a health interview survey, a health examination survey, and a nutrition survey, conducted by specially trained investigators. The survey uses a stratified multistage probability sampling design. Informed written consent for participation was obtained from all study subjects. In addition, the study was approved by the Korea CDC Institutional Review Board. Of the 29,235 Korean participants, 18,997 subjects aged under 50 years were excluded. Participants with diabetes (n = 1,520), renal insufficiency (glomerular filtration rate [GFR] < 45 ml/min/1.73 m^2^, chronic kidney disease [CKD] stage 3b, 4, and 5 according to KDOQI classification [1]) (n = 49), history of treatment for osteoporosis (n = 455), insufficient data (n = 1,613), and fasting time less than 8 hours prior to blood collection (n = 771) were excluded for analysis. Ultimately, 5,830 adults (2,582 men and 3,248 women) were eligible for the present study.

### Definition of MetS and other metabolic status

‘Metabolically obese’ was defined according to the presence of MetS or the highest quartile of IR [13]. For example, individuals within normal BMI range (≥ 18.5 and < 25 kg/m^2^) with (a) the highest quartile of IR estimated by homeostasis model assessment (HOMA) or (b) MetS were defined as MONW [13]. The homeostasis model assessment of insulin resistance (HOMA-IR) is calculated by the formula: fasting glucose (mg/dl) x fasting insulin (uIU/ml) / 405 [14]. According to the National Cholesterol Education Program (NCEP) Adult Treatment Panel III (ATP III) criteria, MetS was diagnosed when the subject had three or more the following criteria: abdominal obesity (waist circumference > 102 cm for men or > 88 cm for women); elevated blood pressure (Systolic blood pressure / Diastolic blood pressure ≥ 130/85 mmHg) or daily use of antihypertensive medication; hyperglycemia (fasting plasma glucose ≥ 110 mg/dl) or current use of insulin or oral medication or a physician’s diagnosis of hypertriglyceridemia (TG ≥ 150 mg/dl) or low HDL cholesterol (HDL cholesterol < 40 mg/dl in men or < 50 mg/dl in women) [15]. The final definition of subtypes of obesity is as follows.

1. Metabolically healthy normal weight (MHNW): non-obese subjects (BMI < 25 kg/m^2^) with the first to third quartiles of HOMA-IR and no presence of MetS.
2. Metabolically healthy obese (MHO): obese subjects (BMI ≥ 25 kg/m^2^) with the first to third quartiles of HOMA-IR and no presence of MetS.
3. Metabolically obese but normal weight (MONW): non-obese subjects (BMI < 25 kg/m^2^) with MetS or with the highest quartile of HOMA-IR.
4. Metabolically obese obese (MOO): obese subjects (BMI ≥ 25 kg/m^2^) with MetS or with the highest quartile of HOMA-IR.

### Measurements

Height and body weight were measured as part of the health examination. BMI was calculated from measured height and weight. Waist circumference was measured to the nearest 0.1 cm at the narrowest point between the lowest rib and the uppermost lateral border of the right iliac crest. Fasting glucose, total cholesterol (TC), triglyceride (TG), LDL cholesterol (LDL-C), HDL cholesterol (HDL-C), PTH, and 25(OH)D were measured from sampled blood collected after overnight fasting. The HOMA estimate of IR was calculated using the formula: fasting blood glucose (mg/dl) × fasting insulin (uIU/ml) / 405. Fasting glucose, TG, and HDL-C were measured enzymatically using a Hitachi automatic analyzer 7600 (Hitachi, Tokyo, Japan). Insulin was measured using a 1470 Wizard Gamma Counter (PerkinElmer, Turku, Finland) with immunoradiometric assay (INS-IRMA kit; Biosource, Nivelles, Belgium). Serum PTH concentration was measured using LIAISON (DiaSorin, Stillwater, MN, USA); serum 25(OH)D concentration was measured using a 1470 Wizard Gamma Counter (PerkinElmer) with a radioimmunoassay (25-Hydroxyvitamin D ^125^I RIA kit; DiaSorin).

### Statistical analysis

Data are expressed as numbers and percentages, or as means ± s.d. As for the parameters without normal distribution, such as PTH, data were expressed as geometric means with 95% confidence interval. Differences between subjects with MetS and those without MetS were evaluated using the Wilcoxon rank sum test or the χ^2^ test, as appropriate. Differences between the four groups according to quartiles of serum 25(OH)D level were determined using a generalized linear model (Duncan’s test of multiple comparisons). Multivariable-adjusted logistic regression analysis was conducted to determine odds ratios (ORs) and 95% confidence intervals (CIs) for the risk of metabolic obesity across quartiles of serum 25(OH)D or PTH levels. Statistical analyses were performed using SAS version 9.3 (SAS Institute, Cary, NC, USA). *P* values of 0.05 were considered statistically significant.

## Results

A total of 5,830 patients were enrolled in this study. The mean age and BMI were 60.7±0.2 years and 23.7±0.1 kg/m^2^ for men and 61.3±0.2 years and 24.1±0.1 kg/m^2^ for women; 36.7% of men and 46.0% of women patients met the criteria for MetS and the prevalence of MetS was significantly different between men and women (*P* < 0.0001). Higher waist circumference, diastolic blood pressure, and fasting triglyceride and glucose levels were observed in men than in women. No significant statistical difference was found in systolic blood pressure between sexes (*P* = 0.0804). HOMA-IR of male participants were lower than the one of female participants (2.04 vs 2.1, *P* = 0.0290). The cut-off values for the highest quartile of HOMA-IR were 2.6 for male and 2.67 for female. Mean serum PTH level was similar between men and women (61.4 [59.9–62.8] pg/mL vs. 62.6 [61.4–63.9] pg/mL, *P* = 0.0903). The difference in serum 25(OH)D concentration between men and women was statistically significant (21.8±0.2 ng/mL and 18.5±0.2 ng/mL, respectively). Vitamin D deficiency, defined as serum 25(OH)D less than 20 ng/ml, was observed in 44.1% of male and 63.9% of female. More detailed baseline characteristics of the study subjects by sex are shown in Table 1.

**Table 1:** Baseline characteristics total population.

	Male (n = 2582)	Female (n = 3248)	P-value
Age (years)	60.7±0.2	61.3±0.2	0.0089
Smoking (%)	34.8	5.1	< .0001
Drinking (%)	17.3	0.9	< .0001
Regular exercise (%)	28.5(1.2)	23.9(1.1)	0.0004
Metabolic syndrome (%)	36.7	46.0	< .0001
BMI (kg m^-2^)	23.7±0.1	24.1±0.1	0.0001
Waist circumference (cm)	84.8±0.2	81.5±0.2	< .0001
Systolic blood pressure (mmHg)	127.0±0.5	125.9±0.5	0.0804
Diastolic blood pressure (mmHg)	81.3±0.3	78.2±0.2	< .0001
Fasting glucose (mg/dl)	97.0±0.3	94.7±0.2	< .0001
Total cholesterol (mg/dl)	188.1±0.8	202.3±0.8	< .0001
Triglyceride (mg/dl)	129.7 (125.9–133.6)	114.9 (112.0–117.9)	< .0001
LDL cholesterol(mg/dl)	113.8±0.8	127.2±0.7	< .0001
HDL cholesterol (mg/dl)	49.0±0.3	53.2±0.3	< .0001
HOMA-IR	2.04 (1.99–2.08)	2.1 (2.06–2.14)	0.0290
PTH (pg/mL)	61.4 (59.9–62.8)	62.6 (61.4–63.9)	0.0903
Serum 25(OH)D (ng/mL)	21.8±0.3	18.5±0.2	< .0001
< 20 ng/mL (%)	44.1	63.9	< .0001
≥20 and < 30 ng/mL (%)	41.5	29.2	
≥30 ng/mL (%)	14.4	6.9	

Abbreviations: BMI, body mass index; LDL, low-density lipoprotein; HDL, high-density lipoprotein; HOMA-IR, homeostasis model assessment of insulin resistance; PTH, parathyroid hormone; Reference range of PTH by provider is 8–76 pg/mL. Data are expressed as the means ± s.e., % (s.e.) or geometric means (95% confidence interval).

Table 2 shows that there were significant differences in PTH levels in women and vitamin D levels in men in MHNW, MONW, MHO, and MOO subjects. Female subjects with metabolic obesity were more likely to have higher levels of PTH than metabolically healthy participants in both crude and adjusted models. In female subjects with metabolically health, PTH level is likely to be lower. Table 2 indicates that female subjects with MHNW had the lowest serum PTH levels, whereas subjects with MOO had the highest serum PTH levels (*P* = 0.0077). In male subjects, differences in serum 25(OH)D levels, but not PTH, were statistically significant according to metabolic status. Male subjects with metabolic health were more likely to have higher serum 25(OH)D levels than metabolically obese subjects in both crude and adjusted models (Table 3). Subjects with MOO had the lowest serum 25(OH)D levels, whereas subjects with MHNW had the highest levels (*P* = 0.0056 in the crude model; *P* = 0.0135 in the adjusted model).

**Table 2:** Comparisons of PTH level according to metabolic status.

	Female		Male	
	Crude	Adjusted^a^	Crude	Adjusted^a^
MHNW	60.6(59.2–62.1)	61.1(59.5–62.7)	61.1(59.4–62.7)	61.0(59.1–62.8)
MHO	63.8(61.5–66.3)	62.9(60.3–65.5)	62.8(60.5–65.3)	63.1(60.5–65.9)
MONW	63.9(60.7–67.3)	64.0(60.9–67.3)	60.2(56.8–63.8)	59.1(55.5–62.8)
MOO	67.9(65.2–70.6)	66.8(64.0–69.7)	61.8(58.2–65.7)	61.5(57.5–65.7)
P-value	< .0001	0.0077	0.4887	0.366

Abbreviations: MHNW, metabolically healthy and normal weight; MONW, metabolically obese but normal weight; MHO, metabolically healthy but obese; MOO, metabolically obese and obese.

^a^ Adjusted for age, BMI, regular exercise, alcohol drinking, smoking, dietary calcium intake, and VitD. Data are expressed as pg/mL (95% confidence interval)

**Table 3:** Comparisons of Vitamin D level according to metabolic status.

	Female		Male	
	Crude	Adjusted^a^	Crude	Adjusted^a^
MHNW	18.6 ± 0.2	18.6 ± 0.3	22.1 ± 0.3	22.5 ± 0.3
MHO	18.4 ± 0.3	18.2 ± 0.4	22.0 ± 0.5	22.0 ± 0.5
MONW	18.2 ± 0.4	18.4 ± 0.4	20.9 ± 0.5	21.3 ± 0.5
MOO	18.2 ± 0.4	18.6 ± 0.4	20.8 ± 0.4	20.8 ± 0.5
P-value	0.6556	0.7812	0.0056	0.0135

Abbreviations: MHNW, metabolically healthy and normal weight; MONW, metabolically obese but normal weight; MHO, metabolically healthy obese; MOO, metabolically obese and obese.

^a^ Adjusted for age, BMI, regular exercise, alcohol drinking, smoking, dietary calcium intake, and PTH. Data are expressed as ng/mL (Estimation ± s.e.)

Table 4 shows that, in normal-weight female subjects, unadjusted ORs for metabolically obese risk were significantly increased with serum PTH levels above a cutoff value of 86.0 pg/mL (OR = 1.764, 95% CI: 1.248–2.494). This association remained unchanged after adjusting for potential confounders, such as age, BMI, physical activity, alcohol drinking habits and dietary calcium intake, PTH and 25(OH)D (OR = 1.678, 95% CI: 1.145–2.460). Serum PTH levels were significantly associated with the risk of metabolic obesity among female but not male subjects.

**Table 4:** Odds ratio for the risk of metabolically obesity expressed in highest quartile of HOMA-IR according to serum PTH concentration.

	Cut off value for serum PTH (pg/mL)	AUC	Odds ratio (95% C.I)	*P*-value
Female (non-obese)	86.0	0.545	1.764 (1.248–2.494) ^a^	0.011
			1.678 (1.145–2.460) ^b^	
Male (non-obese)	51.6	0.511	0.656 (0.469–0.917) ^a^	0.533
			0.587 (0.412–0.835) ^b^	

Abbreviations: HOMA-IR, homeostasis model assessment of insulin resistance.

^a^ Crude value.

^b^ adjusted for age, BMI, regular exercise, alcohol drinking, smoking, dietary calcium intake, PTH and 25(OH)D. Non-obese, BMI ≥ 18, < 25 kg/m^2^; Obese, BMI ≥25 kg/m^2^.

## Discussion

Based on nationally representative survey data, we evaluated the association between serum 25(OH)D and PTH concentrations and the risk of metabolic obesity in a middle-aged and older Korean population with normal to moderately decreased renal function (CKD stages 1 to 3a according to the revised KDIGO classification [1]). Levels of PTH in female subjects and 25(OH)D in males showed a significant association with metabolic health status. In particular, higher PTH was significantly related to increased risk of metabolically unhealthy status even in normal weighted female subjects.

To our knowledge, the present study is the first to investigate the association between serum PTH and 25(OH)D and four subtypes of obesity that represent metabolic and phenotype obesity, namely MHNW, MHO, MONW, and MOO. A strong association between serum PTH concentrations and the risk of metabolic obesity in female subjects was observed in this study. Serum PTH concentrations were significantly higher in female subjects as metabolic obesity and BMI increased. Female subjects with MHNW had the lowest serum PTH levels, whereas female participants with MOO had the highest serum PTH concentrations. In addition, increased risk of metabolic obesity in normal weighted female subjects was found above certain values of PTH. Thus, PTH measurement might have a clinical implication in the evaluation of metabolic status for normal weighted female subjects.

The possible association between PTH and metabolic dysregulation was explained by IR, high blood pressure, hyperglycemia, and dyslipidemia [11]. Research has suggested that the prosclerotic effect of PTH on vascular smooth muscle cells may contribute to vessel wall thickening, which results in elevated blood pressure [16]. However, the exact biochemical mechanisms of how PTH influences metabolic dysregulation have not been established. Our study confirms the positive association between serum PTH and metabolic obesity expressed by IR or MetS in older female subjects, but not in older males. In contrast, Reis et al reported a positive association of PTH with MetS among older men, but not among older women [11,12]. The reason why a positive link between PTH concentration and metabolic obesity appears to be limited to older Korean women cannot be fully explained. It is possible that, together with estrogen deficiency, prevalent vitamin D deficiency due to low dietary calcium consumption among Korean women may influence these findings. Evidence suggests that estrogen acts as a stimulator of intestinal calcium absorption [17,18]. Given that the mean age for female patients was 61.3±0.2 years in our study, most of them could be in menopausal states. Estrogen deficiency may result in the significant elevation of serum PTH concentration to maintain extracellular calcium homeostasis in female subjects. In addition, low dietary calcium intake of dairy calcium has been reported in the Korean population aged ≥ 50 years because of a high prevalence of lactose intolerance and aversion to consume dairy products [19]. In our study, the mean serum 25(OH)D level was significantly lower in female participants compared with males (*P* < 0.001). We speculate that estrogen deficiency combined with low dietary calcium intake resulted in lower serum 25(OH)D concentrations and a subsequent increase of PTH in female subjects. Considering that a number of factors such as age, weight, and waist circumference are related to metabolic dysregulation, we assume that the impact of PTH might appear or be prominent when there is a substantial increase in PTH level. Further research is necessary to confirm our findings and evaluate the mechanisms underlying this association.

In clinical studies, a lower vitamin D level, as assessed by serum 25(OH)D, was associated with the risk of MetS [2,11,20]. In an analysis of NHANES III in the United States, Ford et al indicated a strong inverse relationship between 25(OH)D level and MetS [9]. In their study, abdominal obesity, hypertriglyceridemia, and hyperglycemia significantly decreased as quartiles of serum 25(OH)D levels increased. However, a major limitation of that study was that PTH level was not measured simultaneously and calcium intake was not adjusted. In a nationally representative cross-sectional sample of US adults, Reis et al observed that the strong inverse relationship between 25(OH)D concentration and the risk of MetS was independent of several confounding factors including calcium intake, PTH, BMI, and renal function [11]. One clinical study supports the effect for 25(OH)D in optimizing pancreatic β-cell function. In this study, a negative effect of hypovitaminosis D on β-cell function [21]. However, further studies are required to establish the biological mechanism by which 25(OH)D may influence the development of MetS. Our results also provide support for an inverse relationship between 25(OH)D and the risk of metabolic obesity, and this finding was independent of confounding factors including PTH level and renal function. However, our findings were valid for male subjects, but not for females. Unfortunately, we are unable to explain why the association of 25(OH)D concentration with metabolic obesity appears to be limited to older men. It may be that discrimination of 25(OH)D values according to metabolic status in female subjects was difficult in our study because of the many vitamin D-deficient subjects, as seen in the mean serum 25(OH)D values for female subjects of 18.5±0.2 ng/mL. As recent research on vitamin D revealed that vitamin D3 could be transformed to various metabolites including newly discovered CYP11A1-dependent hydroxyderivatives of vitamin D3, such as 20(OH)D3, 22(OH)D3, 20,22(OH)_2_D3, 20,23(OH)_2_D3, and 1,20(OH)_2_D3 [22,23], evaluation of differences in those metabolites in both gender might be a clue for explanation of the sex-specific findings.

In contrast to previous studies [9,24,25], the present study excluded subjects who had confounding factors that influenced PTH and vitamin D concentrations. First, we adopted a revised classification of CKD and enrolled subjects with CKD stage 1 to 3a only. In 2011, KDIGO proposed a revised classification of CKD [1]. One of the major changes was to subdivide the previously classified CKD 3 stage into two, namely 3a and 3b, depending on GFR. Subjects with stage 3a (GFR 45–59 mL/min/1.73 m^2^) have less risk of vascular complication and mortality compared with the 3b group [26]. Zammit et al suggested that MetS is associated with an increased incidence of stage 3b [27]. Kim analyzed 2,624 Korean adults aged ≥ 50 years and found that serum concentrations of 25(OH)D were significantly lower in subjects with MetS than in those without MetS [10]. Kim proposed that serum 25(OH)D, but not PTH, was an independent risk factor for MetS among middle-aged and older Korean adults [10]. However, a major limitation of that study was the failure to adjust for kidney function. Secondary hyperparathyroidism (SHPT) is a complication of CKD that progresses as GFR decreases [28]. Research has shown that approximately 40% of patients with CKD stage 3 and 80% of patients with stage 4 have SHPT [28]. Research has shown that approximately 40% of patients with CKD stage 3 and 80% of patients with stage 4 have SHPT [28]. Moreover, patients with advanced CKD are advised to take calcium and vitamin D supplements [1]. Therefore, the progression of CKD may influence the changes in both serum PTH and 25(OH)D concentrations. Indeed, Kim reported that the mean serum PTH of the study population was higher than that found in our study (69.9±30.6 pg/mL in subjects with MetS; 68.2±31.8 pg/mL without MetS) [10], which may reflect the fact that participants with advanced CKD were enrolled. Second, factors that could affect PTH and vitamin D levels were corrected. subjects receiving treatment for osteoporosis were also excluded in our study. Although detailed information about medications was not collected, frequently recommended and prescribed medications for osteoporosis including bisphosphonate, calcium, and vitamin D might influence both PTH and vitamin D levels [29]. Additionally, dietary calcium intake was also considered as a confounder in the analysis.

Our study had some limitations. First, we cannot prove causality because of the cross-sectional nature of the data. Second, this study did not consider the amount of sunlight exposure and seasonal variation in the evaluation of 25(OH)D. The average duration of daily sun exposure and seasonal variation need to be adjusted for because the most significant determinant of serum 25(OH)D was the average daily hours of sunlight exposure over at least 2 months prior to the blood sampling [30]. Third, as our findings were observed in a middle-aged and older Korean population, the results of this study may not be generalizable to other age and ethnic groups. Especially, considering the difference in vitamin D metabolism and development of MetS according to ethnicities [31,32], the association of Vitamin D with MetS in our results might be confined or applied to population with prevalent vitamin D deficiency. Fourth, lack of specific data on primary hyperparathyroidism including parathyroid adenoma would be another limitation.

In conclusion, this is the first study to analyze the association between serum 25(OH)D and serum PTH concentrations and the risk of metabolic obesity in a middle-aged and older Korean population with preserved renal function. A positive association between serum PTH concentration and metabolic obesity among female subjects and an inverse relationship between serum 25(OH)D levels and the risk of metabolic obesity were found among male subjects. Further prospective studies are necessary to explore the biological mechanisms underlying these sex-specific findings.
